# Practice and exploration of 24-hour supervision model in personal protection supervision in a COVID-19 isolation ward

**DOI:** 10.1017/ice.2020.391

**Published:** 2020-08-03

**Authors:** Shaxi Li, Yinsheng Guo, Yanqun Zheng, Lin Lei, Xianhu Zeng, Jing Cao, Min Wen, Yingxia Liu, Ting Huang

**Affiliations:** 1 Department of Healthcare-associated Infection Management, The Third People’s Hospital of Shenzhen, Shenzhen, Guangdong, China; 2 Environment and Health Department, Shenzhen Center for Disease Control and Prevention, Shenzhen, Guangdong, China; 3 Nursing Department, The Third People’s Hospital of Shenzhen, Shenzhen, Guangdong, China; 4 Infectious Diseases Department, The Third People’s Hospital of Shenzhen, Shenzhen, Guangdong, China

*To the Editor—*In 2020, the coronavirus disease 2019 (COVID-19) epidemic raged around the world. As of February 12, 1,716 medical staff from medical institutions throughout China had been confirmed as infected.^1–3^ Standard donning and doffing personal protective equipment (PPE) in isolation ward is crucial to reducing the risk of infection. A 24-hour supervision mode was applied in our hospital to maximize the prevention of nosocomial infection by finding and correcting mistakes in a timely way using continuous supervision.

## Methods

From January 11 to February 26, 2020, a video observation mode was used to supervise the donning and doffing process of PPE. When entering the area, staff members did not take identification. Supervisors identified and recorded the errors and provided feedback after the process. Daily observation was conducted between 8:00 a.m. and 5:00 p.m. A 24-hour mode was implemented on February 27, and real-time intercom equipment was added. Identification information of HCWs was collected and a serial number was assigned. If mistakes were observed, they were recorded, and corrections were made in a timely way. Overall, 6 supervisors were assigned to a 24-hour shift after unified training. The supervision contents of the 2 modes were consistent.

### Statistical analysis

We used SPSS version 21.0 software (IBM, Armonk, NY) for statistical analyses. A χ^2^ test was used for statistical analysis among different groups, and logistic regression was used for multivariate analysis. *P* < .05 was considered statistically significant.

## Results

In the video observation mode, 126 observations were recorded and 163 observations were recorded in the 24-hour mode. The PPE doffing procedures were carried out once for each person. In the video observation mode, the error rates of donning and doffing PPE were 2.38% and 12.70%, respectively. The difference was statistically significant (χ^2^ = 61.62; *P* < .001). In the 24-hour mode, the error rates of donning and doffing PPE were 1.22% and 6.0%, respectively. The difference was also statistically significant (χ^2^ = 34.06, *P* < .001). In the 24-hour mode, the total error rate of doffing PPE significantly decreased (χ^2^ = 35.34, *P* < .001). In both modes, the main errors were related to wearing medical protective masks (7.14% vs 5.16%) and hand hygiene (2.38% vs 1.24%). The main mistakes of doffing were related to the removal of disposable protective clothing (32.54% vs 22.56%) and hand hygiene (30.16% vs 19.85%).

We compared the error rates between the 2 groups and analyzed influencing factors in the 24-hour supervision mode. The error rate of HCWs aged >40 years was relatively high (6.44%, χ^2^ = 7.875; *P* = 0.019). The error rate of HCWs was also relatively high (11.52%; *χ* ^2^ = 26.94; *P* < .001). The main factor affecting the error rate was profession. Labor personnel were more likely to make mistakes than were medical staff (Table 1).

**Table 1.** Multifactor Logistic Regression Analysis of Error Rate

Factor	Regression Coefficient	Standard Error	Wald Value	*P* Value	Exp(B)	95% Confidence Interval	
						Upper	Lower
Profession	1.689	0.799	4.473	0.034	5.413	1.132	25.891

## Discussion

In this investigation, the error rate was significantly higher in the PPE doffing procedure than in the PPE donning procedure, which is consistent with the research by Zhang et al^4^ during SARS. The processes of PPE donning and doffing are complicated, especially the doffing process, when the PPE may be contaminated. Therefore, the standardized performance of PPE removal is very important.^5^ After implementation of the 24-hour supervision mode, the error rates of all steps in donning and doffing PPE significantly decreased. One-to-one reminders and timely correction can prevent errors and strengthen the performance of HCWs. Furthermore, HCW awareness regarding risk of infection was raised considerably by these interventions.

During the overall PPE donning and doffing process, errors occurred most frequently while doffing the disposable protective clothing. Doffing action must be gentle, and extremes should be avoided to prevent personal as well as environmental contamination. Secondly, hand hygiene plays an important role in safe donning and doffing. In hand hygiene, the process and rubbing time are equally important. Before donning PPE, hand hygiene must be performed, and only clean hands can touch PPE. When doffing PPE, hand disinfection must be performed for each step and should be carried out again after all PPE has been removed.

In the 24-hour supervision mode, a higher error rate occurred among HCWs aged >40 years than in other age groups. However, no significant difference was detected in the error rate among different professional titles or genders associated with the full extent of training in our hospital in recent years. The major factor influencing the error rate, which is related to the lower education and lack of risk awareness, is the worker population. Nonmedical HCWs are considered a high-risk group for occupational exposure. Improving their risk and self-protection awareness and developing a standard of safe behavior are key issues in future work.

This investigation also has several limitations. The investigation was conducted after the peak period of our domestic epidemic, when the error rate might have been relatively lower than previously. The on-site supervision, video supervision, and intercom reminders were innovatively combined in the 24-hour supervision mode. In addition, the continuous mode provides 24-hour supervision, reminders, and thus, protection for medical personnel. This continuity helps these workers regulate their behavior and reduce risk. Overall, this intervention has proven informative, effective, and successful.
